# Prevalence of Epstein–Barr Virus Infection and Mismatch Repair Protein Deficiency and the Correlation of Immune Markers in Tibetan Patients with Gastric Cancer

**DOI:** 10.1155/2022/2684065

**Published:** 2022-06-13

**Authors:** Jie Shi, Xu Yang, Xinmei Wang, Yufeng Luo, Weixun Zhou, Hanhuan Luo, Zhaxi Bianba, Zhuoma Nima, Qian Wang, Han Wang, Ruiqian Liao, Quzhen Ciren, Mei Li, Junyi Pang

**Affiliations:** ^1^Department of Pathology, Peking Union Medical College Hospital, Chinese Academy of Medical Sciences and Peking Union Medical College, Beijing, China; ^2^Department of Liver Surgery, Peking Union Medical College Hospital, Chinese Academy of Medical Sciences and Peking Union Medical College, Beijing, China; ^3^Department of Pathology, Tibet Autonomous Region People's Hospital, Lhasa, Tibet, China; ^4^Department of General Surgery, Tibet Autonomous Region People's Hospital, Lhasa, Tibet, China

## Abstract

**Background:**

Gastric cancer (GC) is a major cause of cancer-related death in China. Immunotherapies based on PD-1/PD-L1 inhibitors have improved the survival of some patients with GC. Epstein–Barr virus (EBV) infection, mismatch repair (MMR) deficiency, and tumor immune microenvironment (TIME) markers (such as CD3, CD8, and PD-L1) may help to identify specific patients who will respond to PD-1/PD-L1 inhibitors. Considering racial heterogeneity, the pattern of TIME markers in Tibetan patients with GC is still unclear. We aimed to identify the prevalence of EBV infection and the MMR status and their association with immune markers in Tibetan GC to aid in patient selection for immunotherapy.

**Materials and Methods:**

From 2001 to 2015, we retrospectively collected 120 tissue samples from consecutive Tibetan GC patients and constructed tissue microarrays. EBV infection was assessed by Epstein–Barr-encoded RNA (EBER) in situ hybridization, and MMR protein levels were measured. Immune markers (including CD3 and CD8) in intraepithelial, stromal, and total areas were detected by immunohistochemistry (IHC). PD-L1 expression was assessed by the combined positive score (CPS). We also analyzed the relationships of EBV infection and MMR status with immune markers.

**Results:**

Of the 120 samples, 11 (9.17%) were EBV positive (+), and 6 (5%) were MMR deficient (dMMR). PD-L1 CPS ≥1% was found in 32.5% (39/120) of Tibetan GC patients. EBV infection was associated with higher numbers of CD3+ T cells (*P* < 0.05) and CD8+ T cells (*P* < 0.05) and higher PD-L1 expression (*P* < 0.05). For the limited number of dMMR patients, no significant relationship was observed between dMMR and TIME markers (*P* > 0.05).

**Conclusions:**

In Tibetan GC patients, the rates of EBV infection, dMMR, and positive PD-L1 expression were 9.17%, 5%, and 32.5%, respectively. EBV infection was associated with the numbers of CD3+ T cells and CD8+ T cells and PD-L1 expression within the tumor. These markers may guide the selection of Tibetan GC patients for immunotherapy.

## 1. Introduction

Gastric cancer (GC) is the fifth most common cancer and the third most common cause of cancer-related death globally [1, 2]. Based on gene expression profile studies, The Cancer Genome Atlas (TCGA) research network has proposed the following four-tiered molecular classification of GCs: Epstein–Barr virus-positive (EBV+), microsatellite unstable (microsatellite instability high (MSI-high)), genomically stable, and chromosomal unstable [3, 4]. Molecular classification has potential therapeutic implications, particularly stratification according to the EBV and mismatch repair (MMR) statuses [2, 5]. The anti-PD-1 immunotherapies approved to treat GC include nivolumab and pembrolizumab (third line) [6]. EBV positivity, MMR deficiency, and programmed death-ligand 1 (PD-L1) expression are promising biomarkers allowing for the identification of populations most likely to benefit from programmed cell death protein 1 (PD-1)-based immune checkpoint inhibition therapy [6–9]. Moreover, analyzing tumor immune microenvironment (TIME)-based immune infiltrate markers (like CD3, CD8, and PD-L1 expression) may depict the potential mechanism [10–12].

Biological differences between tumors from patients from Eastern and Western countries add to the complexity of identifying standard-of-care therapy based on international trials [2]. Ethnic/racial differences are an important factor in terms of survival and basic characteristics for GC patients [13–15]. Moreover, the genomic and molecular features of GC may vary among ethnicities [16, 17]. In Tibet, which has a unique landscape and different dietary habits (like frequent intake of high-salt diets and dried foods), GC has a high incidence rate [18–20]. With the improvement of living standards and the popularization of new drugs, PD-1 inhibitor therapy may be beneficial for certain Tibetan GC patients. Therefore, it is of great significance to study the potential biomarkers of PD-1 inhibitors in Tibetan GC patients. In this study, we systematically investigated potential biomarkers (EBV, MMR, and PD-L1 status) for PD-1 inhibitor therapy in Tibetan GC patient tissue samples and evaluated their association with the expression of immune markers.

## 2. Materials and Methods

### 2.1. Study Cohorts and Tissue Microarray (TMA) Construction

This retrospective study comprised 120 consecutive patients with stages I–III GC who were treated at Tibet Autonomous Region People's Hospital (Tibet, China) between 2001 and 2015. Patients who received neoadjuvant chemotherapy before surgery and those with inadequate formalin-fixed and paraffin-embedded tissue blocks or TMA cores were excluded from the study.

Representative areas with mixed epithelial tumor tissue and tumor-related stroma were marked on HE-stained slides sampled from TMA blocks. From each sample, 2-6 cores were selected. TMAs with a single 2 mm core per patient were constructed using a TMA instrument. The following clinical data were systematically collected from Tibet Autonomous Region People's Hospital electronic medical records: patient age, sex, histologic grade of differentiation, location, tumor lesion size, differentiation grade, tumor infiltration, lymph node involvement, tumor TNM stage, and vascular invasion. T and N stages were evaluated by the American Joint Committee on Cancer (AJCC) stage version 8 guidelines [21].

The study conformed to the ethical standards set forth in the Declaration of Helsinki and to the national and international guidelines. This retrospective study was approved by the Institutional Review Board of Tibet Autonomous Region People's Hospital (ME-TBHP-21-KJ-054).

### 2.2. EBV In Situ Hybridization (ISH)

EBV infection was tested with an EBV-encoded RNA (EBER) probe (Leica Biosystems) using standard automated methods and batch controls. Cases with tumor cells positive for nuclear EBER were defined as EBV+ GC.

### 2.3. Immunohistochemical Assessment of MMR Proteins, CD3, CD8, and PD-L1

Immunohistochemistry (IHC) analysis was used to detect the MMR-related proteins MSH2, MSH6, MLH1, and PMS2. To assess the TIME, CD3, CD8, and PD-L1 expression were evaluated. IHC was performed using our laboratory protocol as described previously [22, 23]. Briefly, 3-*μ*m-thick TMA serial sections were deparaffinized and subjected to heat-induced epitope retrieval with 10 mM sodium citrate (pH 6.0) at 95°C for 20 min. Endogenous peroxidase activity was quenched using a 0.3% hydrogen peroxide solution.

TMA sections were incubated with primary antibodies against MLH1 (clone ES05, ready to use; Leica Biosystems), PMS2 (clone MOR4G, ready to use; Leica Biosystems), MSH2 (clone 25D12, ready to use; Leica Biosystems), MSH6 (clone PU29, ready to use; Leica Biosystems), CD3 (clone LN10, ready to use; Leica Biosystems), CD8 (clone 4B11, ready to use; Leica Biosystems), and PD-L1 (SP142, 1: 100, ZSGB-BIO, China). Human tonsils treated with primary antibodies were used as positive controls, while the same tissues without primary antibodies comprised the negative controls. After the reactions, all sections were counterstained with hematoxylin. All slides except those used for manual PD-L1 staining were stained using an automatic IHC staining instrument (BOND-III; Leica Biosystems, Wetzlar, Germany) according to the manufacturer's instructions.

### 2.4. Evaluation of Immunostaining

Immunostaining was assessed independently by two pathologists who were blinded to the patients' clinical outcomes. In cases of disagreement, both pathologists reexamined the slides and reached a consensus.

MMR protein loss was considered the complete absence of nuclear staining in tumor cells (TCs) with positive nuclear staining in normal stromal cells and lymphocytes. Tumors were categorized as MMR deficient (dMMR) if the expression of at least 1 MMR protein (MLH1, PMS2, MSH2, and/or MSH6) was lost and as MMR proficient (pMMR) if all 4 MMR proteins had positive nuclear staining in TCs in the presence of an intact internal control.

To assess tumor lymphocyte infiltration markers, an Olympus SZX10 microscope (Olympus Corporation) was used to assess 3-4 independent and intact high-power microscopic areas in each case (magnification, 400× HPF). The most abundant infiltrating lymphocytes were selected, and the numbers of intraepithelial, stromal, and all CD3+ and CD8+ T cells were counted in each microscopic field [24–26]. The average numbers of CD3+ and CD8+ T cells in the selected microscopic fields signified the CD3 and CD8 expression levels, respectively, in each tissue specimen. The number of CD3+ and CD8+ lymphocytes was recorded as a continuous parameter, and using the median as the cutoff, patients were also divided into 2 groups according to the CD3+ and CD8+ T-cell density (high and low). A combined positive score (CPS) ≥1 denoted positive PD-L1 expression. A cutoff of 1 was determined as described in the clinical trials of pembrolizumab in advanced GCs (KEYNOTE-059) [6].

### 2.5. Statistical Analysis

Comparisons of quantitative variables were performed by Student's *t* test and the nonparametric Mann–Whitney/Wilcoxon test, as appropriate. Fisher's exact test was used to evaluate the relationship between EBV status, MMR status, and categorical variables. All statistical analyses were conducted using the Statistical Package for the Social Sciences (version 23; IBM Corp., Armonk, NY). A two-sided *P* value<0.05 was considered statistically significant.

## 3. Results

### 3.1. Clinical Characteristics of Tibetan Patients with GC

A total of 120 consecutive Tibetan patients with GC were included in this study; their median age was 51.5 years (range, 23–74 years). Of these patients, 69.2% were male. The majority of patients (78.3%) had adenocarcinomas; the remaining patients were categorized as follows: 4 (3.3%) had mucinous adenocarcinoma, 5 (4.2%) had signet-ring cell carcinoma, and 17 (14.2%) had mixed pathology type. Overall, 75% of the patients had lymph node metastasis. The TNM stage was I in 13 patients (10.8%), II in 41 patients (34.2%), and III in 66 patients (55.0%).

### 3.2. Clinicopathologic Characteristics of GC Patients according to Their EBV and MMR Statuses

**Figure 1:**
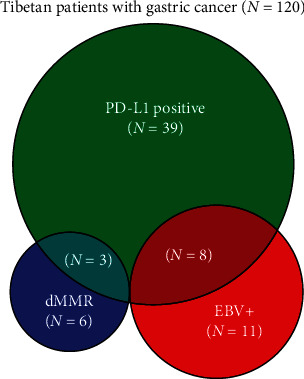
Overlapping EBV-positive, dMMR and PD-L1 expression in 120 Tibetan GC patients in a Venn diagram.

Of 120 Tibetan patients with GC who were assessable by EBER ISH, 11 (9.2%) patients were positive for EBV infection (Figure 1). Regarding MMR status, except for 5 patients who could not be evaluated clearly, only 6 (5.0%) patients were found to have dMMR, while 109 (90.8%) patients had pMMR.

The clinicopathologic characteristics of the patients according to their EBV status and MMR status are shown in Table 1. Univariate analysis revealed that only differentiation (*P* = 0.025) was associated with EBV+ GC (Table 1). No statistically significant associations were observed between the EBV status and MMR status and other clinical and pathological characteristics, such as age, sex, tumor primary site, tumor size, tumor infiltration, TNM stage, and cancer thrombus status (*P* > 0.05).

### 3.3. Immune Marker Landscape in Tibetan Patients with GC

The densities of CD3+ and CD8+ T cells were measured in representative intraepithelial, stromal, and total areas in our Tibetan GC cohort. The median CD3+ lymphocyte infiltration counts in intraepithelial, stromal, and total areas were 107.5, 9.0, and 135.25, respectively, while the median counts of CD8+ lymphocytes in intraepithelial, stromal, and total areas were 81.92, 8.3, and 101.5, respectively. We used the median value as the cutoff to define the high and low infiltration groups.

PD-L1 expression in Tibetan patients with GC was observed in both immune cells and tumor cells, which exhibited a cytoplasmic/membranous staining pattern. Three patients were excluded from the analysis due to IHC failure and thus a lack of PD-L1 expression data. Thirty-nine (32.5%) of the 120 patients exhibited CPS ≥1%, while 65.0% patients were PD-L1 negative. A total of 37.5% of Tibetan GC patients had one of the following characteristics: EBV infection, dMMR, or PD-L1 CPS ≥1%. We also found high overlap of samples with PD-L1 expression with EBV-associated GCs and dMMR GCs; however, EBV-associated GCs and dMMR GCs showed no overlap (Figure 1).

### 3.4. Associations between the EBV Status and Immune Marker (CD3, CD8, and PD-L1) Expression

**Figure 2:**
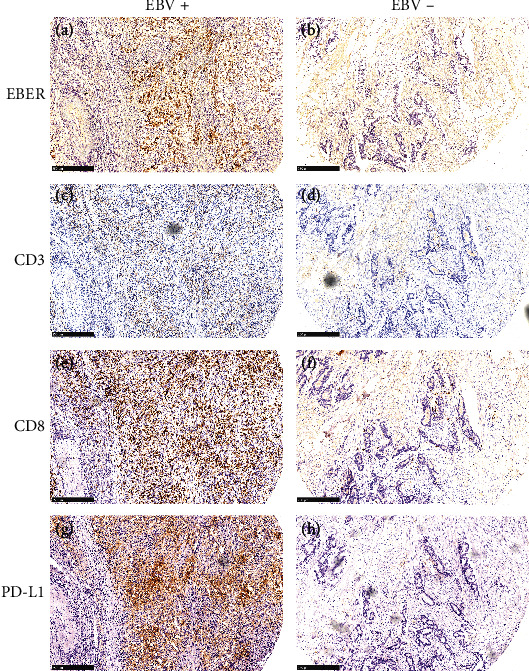
EBV status and representative diagrams of CD3, CD8, and PD-L1 expression. For EBV-positive patients (case 85), EBER status was positive (a), and the expression levels of CD3 (c), CD8 (e), and PD-L1 (g) were high. For EBV-negative patients (case 64), the EBER status was negative (b), and the expression levels of CD3 (d), CD8 (f), and PD-L1 (h) were low. Original magnifications × 200.

The associations between the EBV status and the TIME are presented in Figure 2 as continuous parameters and in Table 2 as categorical variables.

**Figure 3:**
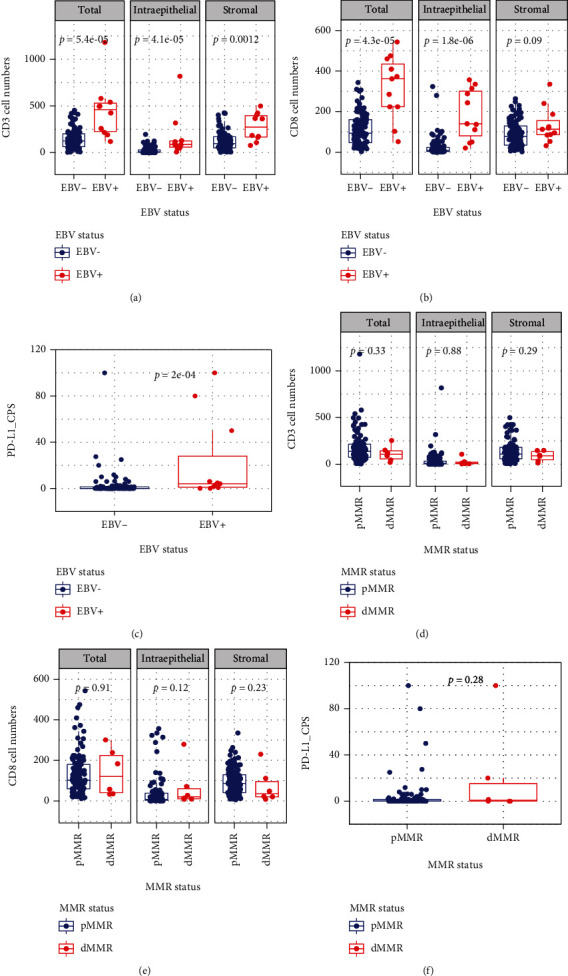
EBV and MMR statuses and PD-L1+, CD3+ and CD8+ cell counts. Analyses of immune markers in EBV+ and EBV− patients revealed that total, intraepithelial and stromal CD3+ (a) and total, intraepithelial CD8+ (b) T-cell lymphocytic infiltrates and PD-L1 expression (c) were more abundant in EBV+ patients than in EBV− patients. However, in patients with different MMR statuses, the differences in the numbers of CD3+ (d) and CD8+ (e) T-cell lymphocytic infiltrates and PD-L1 expression (f) were not statistically significant.

EBV infection in the form of a positive EBER status showed a significantly positive correlation with increased intraepithelial, stromal, and total CD3+ tumor-infiltrating lymphocyte (TIL) counts (Wilcoxon test, *P* = 5.4∗10^−4^, 4.1∗10^−4^, and 0.0012, respectively). EBV infection was also associated with increased intraepithelial (*P* = 1.8∗10^−6^) and total (*P* = 4.3∗10^−5^) CD8+ TIL counts but not with stromal (*P* = 0.09) CD8+ TIL counts. EBV infection was also associated with an increased CPS as a continuous parameter (*P* = 2∗10^−4^) (Figure 3).

Using the median values of CD3+ and CD8+ TIL counts as cutoffs, EBV infection was also associated with increased intraepithelial (*P* = 4.3∗10^−5^), stromal, and CD3+ TIL expression (both *P* = 0.017). We found that EBV infection was also associated with higher intraepithelial (*P* = 0.001) and total (*P* = 0.008) CD8+ TIL counts, while there was still no association with stromal (*P* = 0.053) CD8+ TIL counts. Using CPS ≥1% as a cutoff, EBV infection was also associated with a higher positive PD-L1 expression rate (72.7% vs. 28.4%, *P* = 0.005) (Table 2).

### 3.5. Associations between the MMR Status and the Expression of Immune Markers (CD3, CD8, and PD-L1)

**Figure 4:**
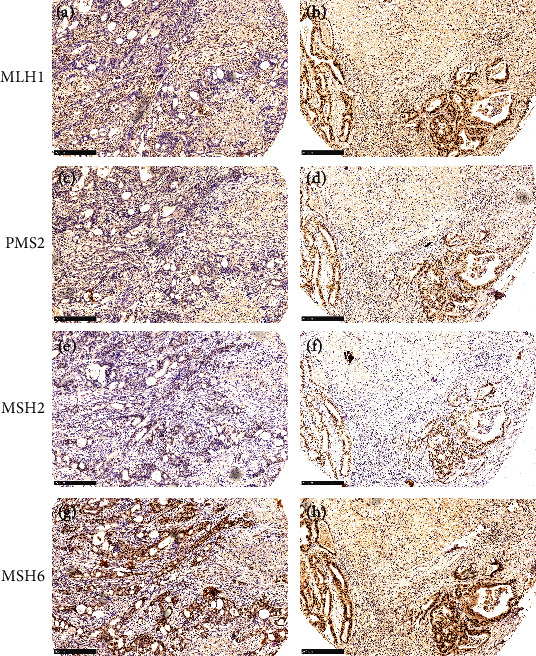
Representative cases of mismatch repair (MMR) deficient (dMMR) and MMR proficient (pMMR). Case 94 of dMMR showed loss of MLH1 (a) and PMS2 (c) but intact expression of the MSH2 (e) and MSH6 (g) proteins. Case 12 of pMMR presented intact expression of the MLH1 (b), PMS2 (d), MSH2 (f), and MSH6 (h) proteins. Original magnifications × 200.

The patterns of dMMR were MLH-1/PMS-2 loss (*N* = 4) and MLH-1/PMS-2/MSH-6 loss (*N* = 2). All six dMMR patients had simultaneous loss of MLH1 (Figure 4 and Table S1). The correlations of the MMR status with CD3, CD8, and PD-L1 expression was then determined. Inconsistent with previously reported studies, we found no statistically significant associations between dMMR (*N* = 6) and the expression of the immune markers CD3, CD8, and PD-L1 when considering the data as either continuous parameters or categorical variables. However, we still observed a trend that patients with dMMR had a higher rate of intraepithelial CD8+ expression (*P* = 0.077) (Figure 3 & Table S2). The sample size of 6 patients with dMMR may have been too small to see significant correlations.

### 3.6. Association of PD-L1 Expression with That of CD3 and CD8

We also used the median values of CD3+ and CD8+ TIL counts as cutoffs, and PD-L1-positive expression was also associated with increased intraepithelial (*P* < 0.001), stromal (*P* = 0.003), and total CD3+ (*P* < 0.001) TIL expression levels. For CD8 expression, we also found that PD-L1-positive expression was associated with higher intraepithelial (*P* = 0.002), stromal (*P* = 0.002), and total (*P* < 0.001) CD8+ TIL expression (Table S3).

## 4. Discussion

Ethnicity is very important for patients with GC. In some genomic and molecular features [16, 17, 27], tumor localization [28, 29] of GC may vary among patients from the West and East. Tibet is located at a high altitude, and the physical and physiological functions of Tibetans have greatly changed to adapt to that environment [30]. GC has become common cancer and needs to analyze in Tibet. To our knowledge, this is the largest study to analyze the molecular classification of Tibetan GC patients. Totally the rate of TNM III stage of Tibetan GC was 55% (66/120), which seem higher than Han patients [31, 32]. Many Tibetan patients maybe not receive standard radical surgery and following systematic therapy after evaluation [20], so new drugs like PD-1 inhibitor and its related biomarker need to investigate. Meanwhile only a subset of patients could benefit from PD-1 inhibitor therapy, so common biomarkers of EBV, dMMR, and PD-L1 need to be investigated to guide the selection of Tibetan GC patients selection for immunotherapy [33]. In our study, 9.17% and 5% of Tibetan GC patients were EBV-positive and dMMR, respectively. PD-L1 CPS ≥1% was found in 32.5% of Tibetan GC patients. EBV infection was associated with higher CD3+ and CD8+ TIL infiltration and higher PD-L1 expression in the TIME.

Previous studies indicated that the prevalence of EBV positivity in patients with GC ranges from 5.1 to 8.4% [3, 34–37], and EBV+ patients with GC always have higher lymphocytic reactions than EBV-negative patients [35, 38–41]. In our 120-patient cohort, the prevalence of EBV positivity was 9.17%. Even though the rate is not very high, but the potential guide of PD-1 inhibitor therapy is important in these patients. Moreover, consistent with previous studies, EBV infection was associated with higher CD3, CD8, and PD-L1 expression. Wang et al. found that EBV+ GC samples had a higher number of CD3+ T cells and higher expression of PD-L1 but not CD8 [40]. Another study found that CD3+ and CD8+ T cells were more abundant in EBV+ GC patients than in EBV− GC patients [10]. Many studies have consistently found that EBV+ GC patients have higher PD-L1 expression [34, 35, 40, 42]. Due to the higher PD-L1 expression and TIL infiltration of EBV+ GC, clinical trials of the PD-1 inhibitor pembrolizumab have achieved a 100% overall response rate (ORR) in 6 patients with EBV+ metastatic GC [7]. Moreover, the ORR was significantly higher for PD-L1+ GC than for PD-L1-negative tumors (50.0% versus 0.0%; *P* < 0.001) [7]. In another small PD-1 treatment GC cohort in Japan, the ORR was 33% [43]. Recently, Bai and colleagues also found EBV+/pMMR could achieve a high ORR and had better survival than EBV-/pMMR patients with GC [44]. Therefore, these findings suggest that EBV+ GC is an “immune hot” subtype and could benefit from PD-1 inhibition.

Many studies have found that the prevalence of dMMR and MSI-H in GC varies from 5.1% to 20.5% GC patients [3, 34, 35, 45, 46]. In our cohort, only 5% of the patients had dMMR tumors. By analyzing TCGA STAD-ESCA data, Zhang et al. found that the dMMR/MSI-H subtype had a higher tumor mutation burden (TMB) but no relationship with the lymphocyte infiltration signature score or CD8+ T-cell abundance [46]. However, Shin et al. found that MSI-H GC patients had higher mean CD3+ and CD8+ T-cell counts but not higher mean CD4+ T-cell counts [11]. For the limited number of dMMR patients in our cohort, no significant relationships between dMMR and TIME markers were observed. dMMR/MSI-H has been confirmed as a biomarker for immune checkpoint inhibitors [47, 48]. In MSI-H GC or gastroesophageal junction cancer patients in the KEYNOTE-059, KEYNOTE-061, and KEYNOTE-062 clinical trials, the ORR was approximately 46.7-57.1% for pembrolizumab and 64.7% for pembrolizumab plus chemotherapy [8]. Kubota et al. also found a 58% ORR for advanced dMMR GC patients with longer progression-free survival (PFS) with anti-PD-1 therapy and a shorter PFS with first-line chemotherapy for advanced GC [43]. In another GC cohort, the PFS of GC patients treated with nivolumab with dMMR was significantly longer than those of patients with pMMR receiving the same treatment [49].

PD-L1 expression is another important biomarker for PD-1/PD-L1 inhibitors [6]. Positive PD-L1 expression was shown to be more common (28.4% vs. 2.7%) in stromal immune cells than in TCs [45, 50]. Liu et al. found that the rate of PD-L1 expression positivity (CPS ≥1) in GC was approximately 59.3% in 300 GC samples [51]. In our cohort, 32.5% of the 120 Tibetan GC patient samples exhibited CPS ≥1%. Many studies have found that positive PD-L1 expression is associated with EBV infection and dMMR and lymphocyte infiltration [7, 51–54]. The KEYNOTE-059 trial found that pembrolizumab can be used as a third-line treatment for patients with low levels of PD-L1 expression (CPS ≥1), and the ORR was 15.5% [6]. However, in the second-line setting (KEYNOTE-061), pembrolizumab did not significantly improve PFS or overall survival (OS) compared with those achieved with paclitaxel in patients with PD-L1+ (CPS ≥1) GC/gastroesophageal junction cancers [55]. The phase 3 KEYNOTE-062 trial compared pembrolizumab with or without chemotherapy versus chemotherapy for the first-line treatment of PD-L1+ (CPS ≥1) GC or gastroesophageal junction adenocarcinoma. Compared with chemotherapy, pembrolizumab was noninferior for OS in patients who had CPS ≥1 but produced fewer adverse events [56]. CheckMate-649 enrolled 1581 GC patients, and first-line nivolumab plus chemotherapy resulted in significant improvements in OS (hazard ratio [HR] 0.71, *P* < 0.0001) and PFS (HR 0.68, *P* < 0.0001) versus chemotherapy alone in patients with a PD-L1 CPS of five or more; moreover, additional results showed that the OS and PFS benefits were retained in patients with CPS ≥1% (HR = 0.77, *P* < 0.0001; HR = 0.74, retrospectively) [57]. Yu et al. also found that MSI-H, EBER, and CPS are meaningful biomarkers for predicting the efficacy of immunotherapy, and combined biomarkers could differentiate better PFS (*P* = 0.01) in patients with GC [58].

A strength of our study is that it included a relatively large Tibetan GC cohort from a single institution. However, there are several limitations that must be considered. First, this was a single-center retrospective study. Second, we did not analyze the molecular and genomic characteristics of this cohort. Third, due to the retrospective nature of the study, we did not have enough prognostic data or translational immunotherapy data. Therefore, further larger and multicenter Tibetan GC patient cohorts should be considered for the analysis of molecular markers and translational immunotherapy efficacy.

In summary, in Tibetan GC patients, the rates of EBV infection, dMMR, and PD-L1 CPS ≥1% were 9.17%, 5% and 32.5%, respectively. EBV infection was associated with the numbers of CD3+ and CD8+ T cells and PD-L1 expression in the TIME. These TIME markers may guide the selection of Tibetan GC patients for immunotherapy.

## Figures and Tables

**Table 1:** Clinical characteristics of Tibetan patients with gastric cancer.

Variable	EBV infection			MMR∗ expression		
	EBV-negative (*N* = 109)	EBV-positive (*N* = 11)	*P* value	pMMR (*N* = 109)	dMMR (*N* = 6)	*P* value
Median age (range)	51 (23-74)	54 (36-62)	0.834	51 (23-72)	59 (54-74)	0.078
Sex—no. (%)			0.092			
Male	78 (71.6)	5 (45.5)		76 (69.7)	4 (66.7)	
Female	31 (28.4)	6 (54.5)		33 (30.3)	2 (66.7)	
Histological type—no. (%)			0.743			0.750
Adenocarcinoma	85 (78.0)	9 (81.8)		84 (77.1)	6 (100)	
Mucinous adenocarcinoma	4 (3.7)	0 (0)		4 (3.7)	0 (0)	
Signet-ring cell carcinoma	4 (3.7)	1 (9.1)		5 (4.6)	0 (0)	
Mixed	16 (14.7)	1 (9.1)		16 (14.7)	0 (0)	
Location— no. (%)			0.225			0.454
Cardia/fundus	8 (7.3)	0 (0)		8 (7.3)	0 (0)	
Gastric body	23 (21.1)	5 (45.5)		28 (25.7)	0 (0)	
Pylorus	78 (71.6)	6 (54.5)		73 (67.0)	6 (100)	
Tumor size—no. (%)			1.000			0.224
<5	44 (40.4)	4 (36.4)		43 (39.4)	4 (66.7)	
≥5	65 (59.6)	7 (63.6)		66 (60.6)	2 (33.3)	
Grade—no. (%)			0.025			0.815
Well differentiation	7 (6.4)	2 (18.2)		9 (8.3)	0 (0)	
Middle differentiation	49 (45.0)	1 (9.1)		44 (40.4)	2 (33.3)	
Poor differentiation	53 (48.6)	8 (72.7)		56 (51.4)	4 (66.7)	
T stage			0.863			1.000
T1	2 (1.8)	0 (0)		2 (1.8)	0 (0)	
T2	19 (17.4)	1 (9.1)		19 (17.4)	1 (16.7)	
T3	58 (53.2)	6 (54.5)		59 (54.1)	3 (50.0)	
T4	30 (27.5)	4 (36.4)		29 (26.6)	2 (33.3)	
N stage			0.792			0.046
N0	27 (24.8)	3 (27.3)		28 (25.7)	1 (16.7)	
N1	22 (20.2)	1 (9.1)		19 (17.4)	4 (66.7)	
N2	20 (18.3)	3 (27.3)		21 (19.3)	0 (0)	
N3	40 (36.7)	4 (36.4)		41 (37.6)	1 (16.7)	
TNM stage (AJCC 8^th^)			0.897			0.843
I	12 (11.0)	1 (9.1)		12 (11.0)	1 (16.7)	
II	38 (34.9)	3 (27.3)		37 (33.9)	2 (33.3)	
III	59 (54.1)	7 (63.6)		60 (55.0)	3 (50.0)	
Vascular invasion			0.458			1.000
Yes	84 (77.1)	7 (63.6)		82 (75.2)	5 (83.3)	
No	25 (22.9)	4 (36.4)		27 (24.8)	1 (16.7)	

Note: ∗Five of 120 patients could not be evaluated for MMR status.

**Table 2:** Correlations between the EBER status and immune microenvironment markers in Tibetan patients with gastric cancer.

Variable	Category	EBV-negative (**N** = 109)	EBV-positive (**N** = 11)	**P** value (Fisher's exact test)
Stromal CD3∗	High	51 (46.8%)	9 (81.8%)	0.017
	Low	58 (53.2%)	1 (9.1%)	

Intraepithelial CD3∗	High	52 (47.7%)	9 (81.8%)	0.017
	Low	57 (52.3%)	1 (9.1%)	

Total CD3_all∗	High	51 (46.8%)	9 (81.8%)	0.017
	Low	58 (53.2%)	1 (9.1%)	

Stromal CD8	High	51 (46.8%)	9 (81.8%)	0.053
	Low	58 (53.2%)	2 (18.2%)	

Intraepithelial CD8	High	49 (45.0%)	11 (100%)	0.001
	Low	60 (55.0%)	0 (0%)	

Total CD8	High	50 (45.9%)	10 (90.9%)	0.008
	Low	59 (54.1%)	1 (9.1%)	

PD-L1 expression^#^	CPS≥1%	31 (28.4%)	8 (72.7%)	0.005
	CPS<1%	75 (68.8%)	3 (27.3%)	

Note: ∗CD3 expression in 1 patient could not be evaluated; ^#^PD-L1 expression in 3 patients could not be evaluated.

## Data Availability

Data are available upon reasonable request.

## Supplementary Materials

Supplementary Materials Table S1: the expression status of each mismatch repair (MMR) protein in Tibetan patients with gastric cancer. Table S2: correlations between the MMR status and immune microenvironment markers in Tibetan patients with gastric cancer. Table S3: correlations between PD-L1 expression and immune microenvironment markers in Tibetan patients with gastric cancer.
