# Supplementary material for: Prevalence of Epstein–Barr Virus Infection and Mismatch Repair Protein Deficiency and the Correlation of Immune Markers in Tibetan Patients with Gastric Cancer
Source: Biomed Res Int. 2022 Jun 13;2022:2684065. doi: 10.1155/2022/2684065 (PMC9208987; doi:10.1155/2022/2684065)
Supplement: Supplementary Materials — Table S1: the expression status of each mismatch repair (MMR) protein in Tibetan patients with gastric cancer. Table S2: correlations between the MMR status and immune microenvironment markers in Tibetan patients with gastric cancer. Table S3: correlations between PD-L1 expression and immune microenvironment markers in Tibetan patients with gastric cancer. [file 2684065.f1.docx]

Table S1. The expression status of each mismatch repair (MMR) protein in Tibetan patients with gastric cancer

|  | **Deficiency** | **Proficient** | **Not evaluated** |
| --- | --- | --- | --- |
| MLH-1 | 6 | 109 | 5 |
| MSH-2 | 0 | 115 | 5 |
| MSH-6 | 2 | 113 | 5 |
| PMS-2 | 6 | 109 | 5 |
| MMR status | 6* | 109 | 5 |

Note: *MLH-1/PMS-2 loss (N=4); MLH-1/PMS-2/MSH-6 loss (N=2).

| Variable | Category | pMMR  (N=109) | dMMR  (N=6) | P value  (Fisher’s exact test) |
| --- | --- | --- | --- | --- |
| Stromal CD3* | High | 55 (50.5%) | 2 (33.3%) | 0.397 |
|  | Low | 53 (48.6%) | 4 (66.7%) |  |
| Intraepithelial CD3* | High | 55 (50.5%) | 3 (50.0%) | 1.000 |
|  | Low | 53 (48.6%) | 3 (50.0%) |  |
| Total CD3* | High | 55 (50.5%) | 2 (33.3%) | 0.679 |
|  | Low | 53 (48.6%) | 4 (66.7%) |  |
| Stromal CD8 | High | 57 (52.3%) | 2 (33.3%) | 0.431 |
|  | Low | 52 (47.7%) | 4 (66.7%) |  |
| Intraepithelial CD8 | High | 52 (47.7%) | 5 (83.3%) | 0.077 |
|  | Low | 57 (52.3%) | 1 (16.7%) |  |
| Total CD8 | High | 56 (51.4%) | 3 (50.0%) | 1.000 |
|  | Low | 53 (48.6%) | 3 (50.0%) |  |
| PD-L1 expression# | CPS≥1% | 34 (31.2%) | 3 (50.0%) | 0.395 |
|  | CPS<1% | 75 (68.8%) | 3 (50.0%) |  |

Table S2. The correlation between MMR status with immune microenvironment markers of Tibetan patients with gastric cancer

Note: *CD3 expression of 1 patient could not be evaluated; #PD-L1 expression of 3 patients could not be evaluated.

Table S3. The correlation between PD-L1 expression with immune microenvironment markers of Tibetan patients with gastric cancer

| Variable | Category | PD-L1 CPS≥1  (N=39) | PD-L1 CPS>=1  (N=78) | P value  (Fisher’s exact test) |
| --- | --- | --- | --- | --- |
| Stromal CD3* | High | 27 (69.2%) | 32 (41.0%) | **0.003** |
|  | Low | 11 (28.2%) | 46 (59.0%) |  |
| Intraepithelial CD3* | High | 29 (74.4%) | 31 (39.7%) | **<0.001** |
|  | Low | 9 (23.1%) | 47 (60.3%) |  |
| Total CD3* | High | 30 (76.9%) | 29 (37.2%) | **<0.001** |
|  | Low | 8 (20.5%) | 49 (62.8%) |  |
| Stromal CD8 | High | 28 (71.8%) | 31 (39.7%) | **0.002** |
|  | Low | 11 (28.2%) | 47 (60.3%) |  |
| Intraepithelial CD8 | High | 28 (71.8%) | 32 (41.0%) | **0.002** |
|  | Low | 11 (28.2%) | 46 (59.0%) |  |
| Total CD8 | High | 31 (79.5%) | 28 (35.9%) | **<0.001** |
|  | Low | 8 (20.5%) | 50 (64.1%) |  |

Note: *CD3 expression of 1 patient could not be evaluated.
